# Identifying copy number variation of the dominant virulence factors *msa* and *p22* within genomes of the fish pathogen *Renibacterium salmoninarum*

**DOI:** 10.1099/mgen.0.000055

**Published:** 2016-04-29

**Authors:** Ola Brynildsrud, Snorre Gulla, Edward J. Feil, Simen Foyn Nørstebø, Linda D. Rhodes

**Affiliations:** ^1^​ Department of Bacteriology and Immunology Lovisenberggata 8, Norwegian Institute of Public Health/Department of Food Safety and Infection Biology, Norwegian University of Life Sciences (NMBU),Oslo,Norway; ^2^​ Department of Bacteriology - Aquatic and Terrestrial Animals, Norwegian Veterinary Institute (NVI), Oslo, Norway; ^3^​ Department of Biology and Biochemistry, University of Bath,Claverton Down, Bath,United Kingdom; ^4^​ Department of Food Safety and Infection Biology, Norwegian University of Life Sciences (NMBU),Oslo,Norway; ^5^​ Northwest Fisheries Science Center, National Marine Fisheries Service, NOAA,Seattle, WA,United States

**Keywords:** copy number variation, gene duplication-amplification, major soluble antigen, p22, renibacterium salmoninarum

## Abstract

*Renibacterium salmoninarum* is the causative agent of bacterial kidney disease, an important disease of farmed and wild salmonid fish worldwide. Despite the wide spatiotemporal distribution of this disease and habitat pressures ranging from the natural environment to aquaculture and rivers to marine environments, little variation has been observed in the *R. salmoninarum* genome. Here we use the coverage depth from genomic sequencing corroborated by real-time quantitative PCR to detect copy number variation (CNV) among the genes of *R. salmoninarum*. CNV was primarily limited to the known dominant virulence factors *msa* and *p22*. Among 68 isolates representing the UK, Norway and North America, the *msa* gene ranged from two to five identical copies and the *p22* gene ranged from one to five copies. CNV for these two genes co-occurred, suggesting they may be functionally linked. Isolates carrying CNV were phylogenetically restricted and originated predominantly from sites in North America, rather than the UK or Norway. Although both phylogenetic relationship and geographical origin were found to correlate with CNV status, geographical origin was a much stronger predictor than phylogeny, suggesting a role for local selection pressures in the repeated emergence and maintenance of this trait.

## Data Summary

1. The sequence data for all isolates used in this study is available for download from http://trace.ncbi.nlm.nih.gov/Traces/sra/?study=ERP003780

## Impact Statement

This article identifies expansive duplication of the genes encoding the dominant virulence factors *msa* and *p22* in the fish pathogen *Renibacterium salmoninarum*, the organism responsible for bacterial kidney disease of salmonid fish. *R. salmoninarum* is a highly clonal bacterium with a very limited accessory genome, and although duplication of *msa* is already known as a concept, this study extends the finding to *p22*, the other major surface protein. The number of identical gene copies may in some cases be as high as five. The data suggest multiple independent duplication events that appear to be much more common in strains circulating in the Pacific Northwest region of North America, pointing to local selection pressures as important for the repeated emergence.

Gene copy number variation in bacteria is probably severely underreported, and there are very few reports on the regional distribution of the phenomenon. It is hoped that the findings and methodology presented in this article may serve to fuel the interest in performing gene copy number variation studies, as the mechanism is increasingly being seen as more frequent and phenotypically important than previously believed.

## Introduction

*Renibacterium salmoninarum* is the causative agent of bacterial kidney disease (BKD) in cultured and wild salmonid fish. BKD can result in acute morbidity or mortality, or it can be a slowly progressive disease causing an often dramatic decline in growth. BKD is economically important in aquaculture, where it can spread horizontally throughout sea pens of juvenile and subadult Atlantic salmon (*Salmo salar*) (Murray *et al.*, 2012) or vertically through transferred broodstock or eggs (Evelyn *et al.*, 1986). It is also a concern for conservation and restoration efforts for endangered fish stocks because infections are prevalent among more susceptible free-ranging Pacific salmon in river and marine systems (Pascho *et al.*, 1993; Rhodes *et al.*, 2011; Sandell *et al.*, 2015).

Although the pathogenicity of *R. salmoninarum* is incompletely understood, several antigenic determinants have been described, including the dominant immunogenic protein major soluble antigen (MSA) (Turaga *et al.*, 1987; Wiens and Kaattari, 1991), an abundant heat-stable 57 kDa extracellular protein that makes up 60–70 % of all surface proteins in *R. salmoninarum* (Fredriksen *et al.*, 1997; Wood and Kaattari, 1996), and is involved in immunosuppression (Brown *et al.*, 1996; Fredriksen *et al.*, 1997; Turaga *et al.*, 1987), agglutination (Senson & Stevenson, 1999; Wiens *et al.*, 1999; Wiens and Kaattari, 1991) and virulence (Coady *et al.*, 2006; O’Farrell & Strom, 1999; Senson & Stevenson, 1999). Other antigenic determinants include capsular synthesis, heme acquisition operons, haemolysins and an immunosuppressive 22 kDa surface protein provisionally named *p22* (Fredriksen *et al.*, 1997). The *p22* gene encodes a poorly described loosely associated surface protein (Fredriksen & Bakken, 1994) that has been implicated in suppression of antibody production and a stronger agglutination of leucocytes than that which is seen for the MSA protein (Fredriksen *et al.*, 1997).

The genome of the type strain of *R. salmoninarum*, ATCC 33209^T^, contains two identical transcriptionally active copies of the MSA-encoding gene: *msa1* and *msa2* (O’Farrell & Strom, 1999; Rhodes *et al.*, 2002). Both genes are essential for the development of clinical disease and mortality (Coady *et al.*, 2006). Whilst it seems certain that a single copy was originally acquired through horizontal gene transfer and subsequently duplicated within the bacterial genome (Wiens *et al.*, 2008), the origin of this gene is unclear, as no homologue to the *msa* gene has ever been found in any other sequenced genome. Both *msa* loci are flanked by insertion sequences and transposases, and *msa2* is additionally flanked by several degraded genes related to conjugation (including *traA* relaxase, type IV secretion protein and site-specific recombinase resolvase). Because multiple copies of identical genes are unusual in bacterial genomes, O’Farrell & Strom (1999) suggested that multiple *msa* copies might confer a selective advantage. Subsequently, Rhodes *et al.* (2004) demonstrated the presence of a third copy in some isolates, and provided clear evidence for a positive correlation between *msa* copy number and mortality at lower infection doses.

The gene content variation of this species appears to be exceptionally low, with core- and pan-genomes reported to be very similar even for strains sampled over 50 years from a wide range of habitats (Brynildsrud *et al.*, 2014). However, this does not include paralogues, and the findings of Rhodes *et al.* (2004) suggest that copy number variation (CNV) in the *msa* genes of *R. salmoninarum* has phenotypic relevance. Gene duplication has been shown to be adaptive in bacteria (Riehle *et al.*, 2001), and CNV is known to be an important mechanism for dose variation of specific proteins under appropriate environmental conditions (Stranger *et al.*, 2007). As an example, a recent study demonstrated that some strains of *Mycobacterium tuberculosis* harboured a large, tandem gene duplication and noted greater expression of an anaerobic survival regulon that is contained within the duplication (Domenech *et al.*, 2010).

The aim of the present study was to screen a diverse collection of *R. salmoninarum* isolates for evidence of CNV in any of the core genes and, if found, to investigate phylogenetic and spatial patterns of the distribution of genetic variants. This work can provide a better understanding of *Renibacterium* microevolution and may shed light on the mechanisms of differential disease manifestation in different populations.

## Methods

### Computational analyses

Sixty-eight isolates whose spatial and temporal origins varied widely were sequenced on an Illumina GAII platform at The Genome Analysis Centre (TGAC), Norwich, UK, as part of a previous effort by the authors, and are available at the Sequence Read Archive of the National Center for Biotechnology Information (NCBI) under the accession numbers listed in Table 1. Non-pairing reads, reads containing ambiguous characters and reads with an average PHRED score of <20 were discarded before alignment to reference genome ATCC 33209 (available from NCBI GenBank under accession number NC010168) with Geneious v7.1 (Biomatters), using the option to randomly map reads with multiple best hits.

**Table 1.** *R. salmoninarum* isolates screened for copy number variation

Sample ID	Host	sw/fw	f/w	Origin	Year	Alternative ID	EBI accession no.
MT1351	*S. salar*	sw	f	Scottish Highlands, UK	1993		ERR327904
Carson 5b†	*O. tshawytscha*	fw	f	Tyee Creek/Wind River, USA	1994		ERR327905
05372K*	*O. tshawytscha*	sw	f	Grande Ronde Basin, USA	2005		ERR327906
NCIMB 1116	*S. salar*	fw	w	River Dee, UK	1962	96056	ERR327907
NCIMB 1114	*S. salar*	fw	w	River Dee, UK	1962	5005	ERR327908
MT1880	*S. salar*	sw	f	Strathclyde, UK	1996		ERR327909
MT1470	*O. mykiss*	fw	f	Tayside, UK	1994		ERR327910
NCIMB 2235	*O. tshawytscha*	sw	f	Oregon, USA	1974	ATCC 33209	ERR327911
9025	*O. mykiss*	fw	f	Yorkshire, UK	2009	16251-1	ERR327912
MT239	*S. salar*			Scotland, UK	1988		ERR327913
MT1511	*O. mykiss*	fw	f	Strathclyde, UK	1994		ERR327914
Cow-chs-94*	*O. tshawytscha*	fw		Cowlitz River, USA	1994	GR 16	ERR327915
MT444	*S. salar*	sw	f	Western Isles, UK	1988		ERR327916
MT839	*S. salar*	sw	f	Scottish Highlands, UK	1990		ERR327917
MT452	*O. mykiss*	fw	f	Dumfries and Galloway, UK	1988		ERR327918
MT861	*S. salar*	sw	f	Scotland, UK	1990		ERR327919
MT1363	*O. mykiss*	sw	f	Strathclyde, UK	1993		ERR327920
99333	*O. mykiss*	fw	f	Wales, UK	1998	980036-102	ERR327921
MT1262	*S. salar*	fw	f	Scottish Highlands, UK	1992		ERR327922
5007	*O. mykiss*			Scotland, UK	2005	0180-18	ERR327923
MT3313	*O. mykiss*	fw	f	Central Scotland, UK	2008		ERR327925
MT3277	*O. mykiss*	fw	f	Dumfries and Galloway, UK	2008		ERR327926
96071	*O. mykiss*	fw	f	Hampshire, UK	1996	TEST VALLEY FDL	ERR327927
MT3315	*O. mykiss*	fw	f	Strathclyde, UK	2008		ERR327928
MT2622	*O. mykiss*	sw	f	Strathclyde, UK	2002		ERR327929
1205	*O. mykiss*		f	UK	2001	3104-67	ERR327930
99327	*O. mykiss*	fw	f	UK	1997	970313-2	ERR327931
7105	*O. mykiss*		f	UK	2007	P0416 T83 10-3 2	ERR327932
MT3479	*S. salar*	sw	f	Orkney, UK	2008		ERR327933
MT3482	*S. salar*	sw	f	Strathclyde, UK	2009		ERR327934
MT2979	*O. mykiss*	fw	f	Scottish Highlands, UK	2005		ERR327935
MT2943	*S. salar*	sw	f	Scottish Highlands, UK	2005		ERR327936
99329	*O. mykiss*	fw	f	Wales, UK	1998	980036-125	ERR327937
99326	*O. mykiss*	fw	f	Wales, UK	1999	2119-8	ERR327938
MT3106	*O. mykiss*	fw	f	Strathclyde, UK	2006		ERR327939
99344	*O. mykiss*	fw	f	Hampshire, UK	1998	980106-1.1.5	ERR327940
MT3483	*S. salar*	sw	f	Strathclyde, UK	2009		ERR327941
5006†	*O. kisutch*	sw	f	Bella Bella, Canada	1996	960046	ERR327942
99332	*O. mykiss*	fw	f	Wales, UK	1999	2119-3	ERR327943
Rs 8	*S. salar*	sw	f	New Brunswick, Canada	2008		ERR327944
Rs 10*	*S. salar*	sw	f	New Brunswick, Canada	2009		ERR327945
Rs 4	*S. salar*	sw	f	New Brunswick, Canada	2006		ERR327946
Rs 3	*S. salar*	fw	f	New Brunswick, Canada	2005		ERR327947
99345	*O. mykiss*	fw	f	Wales, UK	1998	980070-18	ERR327948
99341	*O. mykiss*	fw	f	Hampshire, UK	1998	980109-20	ERR327949
Rs 5	*S. salar*	sw	f	New Brunswick, Canada	2007		ERR327950
Rs 2*	*S. salar*	sw	f	New Brunswick, Canada	2005		ERR327951
BPS 91*	*O. gorbuscha*			Nanaimo, Canada	1991		ERR327952
Rs 6*	*S. salar*	sw	f	New Brunswick, Canada	2007		ERR327953
DR143	*S. fontinalis*	fw	w	Alberta, Canada	1972	GR 17	ERR327954
6553	*S. salar*	sw	f	Hemne, Norway	2008	2008-09-495	ERR327955
6642	*S. salar*		f	Hemne, Norway	2008	2008-06-633	ERR327956
Car 96	*O. tshawytscha*			Washington, USA	1996		ERR327957
684	*S. trutta*	fw	f	Aurland, Norway	1987		ERR327958
GR5*	*T. thymallus*	fw	w	Montana, USA	1997	980036-87	ERR327959
WR99 c2	*O. kisutch*			Washington, USA	1999		ERR327960
D6	*O. tshawytscha*			Oregon, USA	1982		ERR327961
6694	*O. mykiss*	sw	f	Hemne, Norway	2008		ERR327962
BQ96 91-1*	*O. kisutch*			Nanaimo, Canada	1996		ERR327963
5223*	*S. salar*	sw	f	Kvinnherad, Norway	2005	2005-50-579	ERR327964
6863	*O. mykiss*	sw	f	Osterøy, Norway	2009		ERR327965
7441	*S. salar*		f	Storfjord, Norway	1985	1985-09-667	ERR327966
7450	*S. salar*		f	Askøy, Norway	1987	1987-09-1185	ERR327967
6695	*O. mykiss*	sw	f	Hemne, Norway	2008	2008-06-631	ERR327968
7449	*S. salar*		f	Skjervøy, Norway	1987	1987-09-932	ERR327969
7448	*S. salar*		f	Stranda, Norway	1986	1986-09-4366	ERR327970
7439	*S. salar*		f	Sognefjorden, Norway	1984	1984-40.992	ERR327971
5004	Unknown			USA	1960s	NCIMB 1111	ERR327924
ATCC 33209‡	*O. tshawytscha*	sw	f	Oregon, USA	1974		NC_010168.1

sw/fw saltwater/freshwater habitat; f/w farmed/wild fish origin.

*Duplication in *msa-p22*k.

†Other gene duplication.

**‡**Type strain. Sequence data downloaded from Genbank.

CNVs were discovered using the R (R Development Core Team, 2012) package *CNOGpro* (Brynildsrud *et al.*, 2015) with the following parameters: coverage counted in sliding windows of length 50 bp, prior probability of changing states (for each read count observation) was set to p=1.0×10^–10^ and the error-rate parameter was set to 0.01. The *runHMM* method was used to call CNV regions and copy numbers were considered correct if they agreed with credible intervals (percentiles 1–99) from the *runBootstrap* method. When evaluating results we discarded IS*994* tallies, as 69 copies (69 *orfA* and 67 *orfB*) of this element are known to exist in the reference genome (Wiens *et al.*, 2008), making it impossible to evaluate copy number variation with our method. We also considered standalone CNV calls in segments shorter than 300 bp as unreliable, as such calls could happen from chance alone (Brynildsrud *et al.*, 2015). When quantifying total *msa* enrichment, the signal from *msa1* and *msa2* were added together, and the relative frequencies were inferred by inspecting the signal from the hypothetical protein-encoding gene *p12* (Fig. S1, available in the online Supplementary Material). These results were corroborated using real-time quantitative PCR (qPCR) on selected isolates with different copy number multiplicities (detailed in the Supplementary Material).

**Fig. 1.**
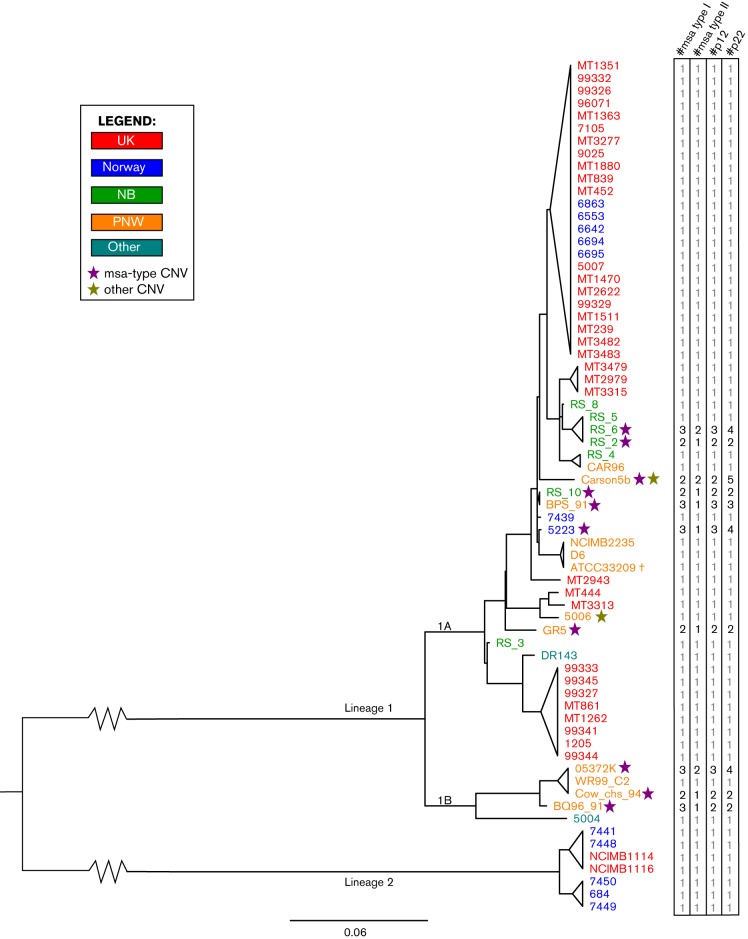
CNV distribution by phylogeny and geography. Phylogenetic tree revealing patterns of CNV distribution. Horizontal branches represent patristic distances, and isolates are coloured according to their origin. Purple stars indicate CNV in the *msa* and *p22* genes, while an olive star represents CNV in other genes. The most probable copy number of each of the *msa* (types I and II), *p12* and *p22* genes is shown on the extreme right. ATCC 33209 is marked with double dagger symbol because it is a duplicate of NCIMB 2235 and thus not counted separately when tallying CNV frequencies. The inter-lineage distance has been truncated and represents one third of the actual distance. ‘NB’ represents isolates from New Brunswick, Canada, and ‘PNW’ represents isolates from British Columbia, Canada, as well as Washington, Oregon and Montana, all USA. The isolate with unknown North American origin and the one from Alberta, Canada, has been labelled ‘Other’. Adapted from Brynildsrud *et al.* (2014).

### Regression analysis

The presence or absence of *msa* CNV in an isolate was considered a binary trait, and associations between this trait and year of isolation, host species and saltwater/freshwater habitat were investigated by logistic regression, both bivariable and multivariable with interaction terms using R.

### Cluster analysis through matrix correlation

Phylogenetic trees were created from single nucleotide polymorphism alignments with the program MrBayes (Ronquist & Huelsenbeck, 2003) (see Supplementary Material). Pairwise patristic distances between isolates were calculated as the sum of branch lengths between leaf pairs of the consensus tree. Pairwise geodesic distances between isolates' geographical origins were calculated by solving for central angle in the spherical law of cosines and multiplying by the radius of the Earth. The latitude–longitude coordinates were rounded to the nearest degree. In some cases the exact sample origin was not known, so the coordinate pair was set to represent geographical midpoints for the sub-national region. To test for phylogenetic and spatial clustering of CNV presence/absence, we created a binary matrix where equal CNV statuses of isolate pairs were coded as 1 and unequal as 0. In this analysis we regarded isolates with asterisks listed in Table 1 as positive for the duplication in question and the remaining isolates as negative. We then adopted a Mantel test-like approach by performing the Mann–Whitney U test of equal distributions between groups defined by CNV status on patristic/geodesic distance data. This test estimator was subsequently compared with those obtained from 10 000 random permutations of the CNV status matrix. The trait was considered to be phylogenetically or spatially clustered if the test estimator fell below the lower 1-percentile limit in the distribution of permuted data set estimators.

## Results

Overall, very little CNV was seen in our isolates. In fact, the coverage data of most isolates (57/68) indicated no variation at all. This finding is consistent with previous reports of a high degree of sequence conservation in the *R. salmoninarum* genome. Nevertheless, CNV was found in 11 isolates, shown in Fig. 1.

A complete list of all CNVs discovered in this study can be found in Table 2. In total, there were nine distinct CNV regions. Four of these were unique to the Carson5b isolate and two to isolate 5006. The remaining CNVs were non-unique and occurred jointly (i.e. the presence of one CNV type also implied the presence of the others) in all 11 CNV isolates. Among these were duplications of the genes encoding the primary surface surface proteins *of R. salmoninarum*: the *msa* gene and a 22 kDa hypothetical protein (hereafter referred to as *p22*).

**Table 2.** Copy number estimates in CNV isolates. Duplicated genes with the copy number and 95% confidence intervals (95% CI). The most probable copy number is based on the most common local copy number state from the Hidden Markov Model method. The individual *msa* gene copy numbers could not be differentiated and have been merged. All results are from CNOGpro (Brynildsrud *et al*., 2015)

Isolate	Gene	Copy number	95% CI	Most probable
5006	*msa1*	1.1	1.0–1.2	2
*msa*2	1.2	1.1–1.3
*p*1*2*	0.8	0.6–1.0	1
*p22*	1.2	1.0–1.4	1
2 974 628 to 3 084 569 (segmental duplication)	1.4	1.4–1.4	2
3 088 016 to 3 100 482 (segmental duplication)	1.6	1.5–1.6	2
5223	*msa1*	2.3	2.1–2.4	4
*msa2*	2.1	2.0–2.3
*p12*	2.8	2.4–3.1	3
*p22*	3.8	3.3–4.3	4
05372K	*msa1*	2.5	2.3–2.6	5
*msa2*	2.4	2.2–2.7
*p12*	2.9	2.5–3.3	3
*p22*	4.3	3.9–4.7	4
BQ96_91	*msa1*	1.8	1.7–1.9	4
*msa2*	1.8	1.6–1.9
*p12*	2.9	2.5–3.3	3
*p22*	1.8	1.4–2.1	2
BPS91	*msa1*	2.0	1.9–2.1	4
*msa2*	2.1	1.9–2.3
*p12*	2.5	2.4–2.8	2
*p22*	2.6	2.4–2.8	3
Carson5b	*msa1*	2.1	1.9–2.4	4
*msa2*	2.1	1.9–2.4
*p12*	2.1	1.7–2.7	2
*p22*	5.3	4.2–6.4	5
Rsal33209_0109 (lacI family trans. reg.)	1.6	1.2–2.1	2
Rsal33209_1458 (NADH-dep. flav. oxidored.)	1.4	1.0–2.0	2
Rsal33209_2607 (ferredox. NADH red.)	1.6	1.3–2.0	2
Rsal33209_3193 (hypothetical protein)	1.9	1.4–2.4	2
Cow-Chs-94	*msa1*	1.7	1.5–1.8	3
*msa2*	1.6	1.5–1.8
*p12*	1.6	1.3–2.0	2
*p22*	2.2	1.9–2.5	2
GR5	*msa1*	1.4	1.2–1.5	3
*msa2*	1.4	1.3–1.5
*p12*	2.4	2.3–2.6	2
*p22*	1.9	1.7–2.0	2
RS2	*msa1*	1.5	1.4–1.7	3
*msa2*	1.5	1.4–1.7
*p12*	2.2	2.0–2.3	2
*p22*	1.7	1.3–2.1	2
RS6	*msa1*	2.4	2.2–2.7	5
*msa2*	2.5	2.3–2.7
*p12*	2.6	2.0–3.1	3
*p22*	3.7	3.4–4.0	4
RS10	*msa1*	1.6	1.5–1.7	3
*msa2*	1.5	1.4–1.7
*p12*	1.9	1.7–2.2	2
*p22*	1.9	1.7–2.2	2

The total number of *msa* copies in CNV-positive isolates ranged from two to five. This confirms the supposition that the minimum copy number of *msa* genes is two, as no isolate presented a read coverage that was suggestive of only a single copy. There were two different *msa* duplication types, for which we provisionally introduce the nomenclature ‘type I’ and ‘type II’. Type II was a subunit of type I, but the two can be differentiated by type II’s lack of a marker gene, *p12* (a predicted gene annotated Rsal33209_1032). As this gene is only part of type I duplications, the relative frequency of the two types can be found by inspecting the coverage of the *p12* gene (Fig. S1).

Type I *msa* duplication included the *msa* gene, the *p12* marker gene, the transposase-encoding Rsal33209_0133 and the inactivated insertion sequence (IS) sequence IS*Rs3*, including all intergenic segments and flanking inverted IS*994* sequences. Type I *msa* duplication thus very closely resembles the genomic region roughly between coordinates 110 000 and 115 000 in ATCC 33209, and is surely a duplication of the *msa1* gene.

**Fig. 2.**
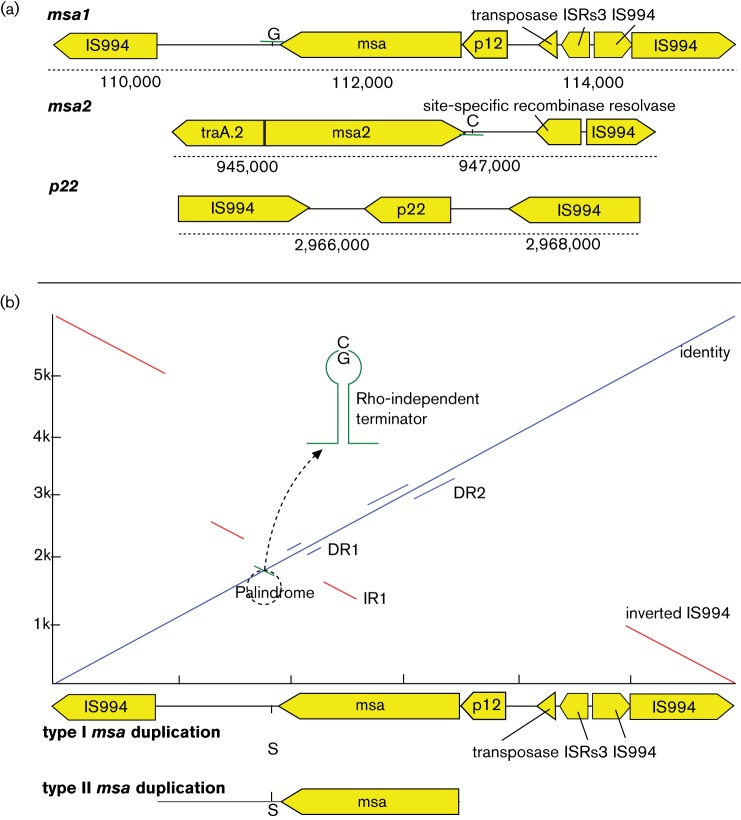
Duplication maps with gene dotplot. (a) Schematic view of the three major CNV regions discovered in the current study. (b) Genome dot plot of the major (type I) and minor (type II) *msa* duplication units to itself, showing repeat regions and palindromic sequence. Solid lines represent a minimum of 85% sequence identity. DR, direct repeat; IR, inverted repeat. The 91 bp palindrome encodes a predicted rho-independent terminator with a central loop polymorphism between *msa1* and *msa2*. The polymorphism is located 37 bp downstream of the *msa* ORF. We could not resolve the correct orientation of this segment in duplications, and the polymorphism is therefore labelled by the ambiguity character S (C/G).

Type II *msa* duplication included the *msa* gene with the intergenic sequence from the terminus of the gene and roughly 800 bp downstream, which resembles two different regions of ATCC 33209: coordinates 110 400 to 112 901 or 945 077 to 947 575 in ATCC 33209. We could therefore not determine whether type II duplications represent duplications of *msa1* or *msa2*, and unfortunately read mapping proved unhelpful to investigate this. Although the *msa1* and *msa2* genes differ very slightly at upstream and downstream sites, the ORFs themselves are identical, and there are several large (130–180 bp) inverted and direct repeats plus one 91 bp perfect palindrome associated with the gene, confounding read mapping (Fig. 2). However, previous experiments have only found duplications of *msa1* (Rhodes *et al.*, 2004). An *msa1* origin must also be suspected for our data due to the fact that the *traA.2* gene neighbouring *msa2* was not duplicated in any isolates.

It remains unknown whether *msa* loci are differentially regulated. Using the terminator prediction tool ARNold (Naville *et al.*, 2011) and the RibEx riboswitch explorer (Abreu-Goodger & Merino, 2005), we discovered that the palindrome at the 3' of the *msa* ORF contained a predicted rho-independent terminator/riboswitch-like element at both the *msa* loci, although with ‘G’ as the central loop nucleotide for *msa1* and ‘C’ for *msa2*, opening the possibility for riboswitch-mediated regulation (Fig. 2).

The third non-unique CNV region matched the region between coordinates 2 965 759 and 2 967 751 in ATCC 33209. This region is flanked by inverted IS elements and contains a single ORF, encoding the *p22* protein (a 22 kDa hypothetical protein labelled RSal33209_3334). Also part of the duplication unit was the intergenic segments on both sides of this ORF. The total number of *p22* copies in *msa*-duplicated isolates was estimated as ranging from two to five.

### Trait clustering

**Fig. 3.**
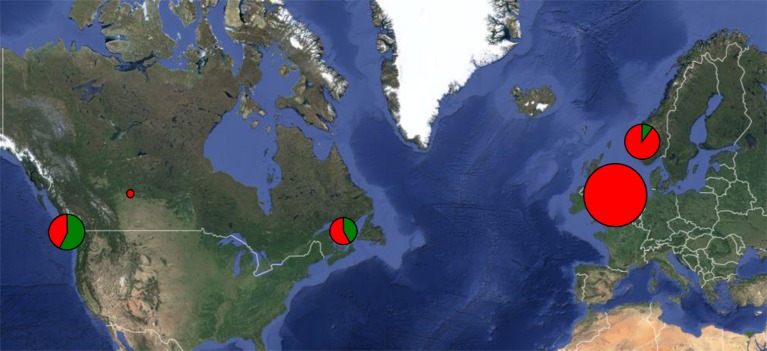
Geographical distribution of CNV isolates. Relative frequencies of CNV-positive isolates from each major sample region. Isolate origin has been truncated down to represent either Norwegian, UK, New Brunswick and Pacific Northwest (including the Canadian province of British Columbia, as well as the US states of Washington, Oregon and Montana), except for a single isolate from Alberta, Canada. At each location, the size of the pie chart represents the number of isolates. The red sectors and green sectors indicate the fraction of CNV-negative and CNV-positive isolates, respectively.

The *msa–p22* duplication trait did not correlate with year of isolation, host species or saltwater/freshwater habitat. However, a strong geographical pattern was seen in the presence of gene duplication. CNV was absent in the exclusively European lineage 2 (lineage notation from Brynildsrud *et al.*, 2014), and limited to defined clusters within the widely distributed lineage 1A and the Pacific Northwest-associated lineage 1B. Among the 10 isolates containing additional copies of *msa* and *p22* genes, six are from the Pacific Northwest, three are from Eastern North America (New Brunswick, Canada) and oneis from Norway, corresponding to 55, 43 and 8% of the total investigated isolates from each respective region. Notably, of the 36 UK isolates, not one displayed CNV (Fig. 3).

**Fig. 4.**
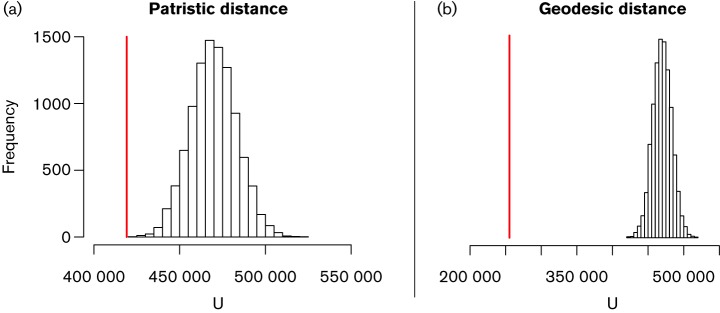
Mantel correlation between CNV and phylogeny/geography. Mann–Whitney U test statistic distribution in the Mantel correlation analysis. Correlation is measured between pairwise patristic (a) and geodesic (b) distances to identical CNV status, measured as a binary trait. The vertical red line represents our observed statistic and the white boxes represent the histogram of the 10 000 permuted matrix-statistics. Note the Gaussian distribution of U values for both the patristic (a) and the geodesic (b) distance analyses. The increased distance between our observed U and the permuted matrix-U valuess in (b) indicates a more extreme correlation.

To test whether CNV was clustered within different phylogenetically and spatially defined groups, we used Mantel correlation analyses (Fig. 1). For geodesic data, we found the Mann–Whitney U estimator to be 255 344, compared with the full range 432 002–515 531 from the permuted dataset, which translates to a p-value of <1.0×10^–4^ when calculated conservatively as in Diniz-Filho *et al.* (2013). However, because the distribution of U values follows a near-perfect normal distribution (as calculated by the Anderson–Darling test of normality), a parametric p-value estimation of p<1.0×10^–50^ can be used (Fig. 4). In other words, CNV was strongly clustered into geographically defined groups. This can also happen because phylogenetically related isolates tend be spatially clustered, so we also investigated whether the pairwise patristic distances between isolates impacted the CNV. For these data, Mann–Whitney's U was computed as 419 090, which is also lower than the full range of all permuted-matrix values (424 489–525 215) (non-parametric p<1.0×10^–4^; Gaussian parameterization p=7.4×10^–5^). Although this implies association between patristic distance and CNV as well, the pairwise geodesic distance is a much stronger predictor of CNV status, implying that these gene duplications are primarily the result of local selection pressures.

## Discussion

Although duplicates of genes encoding ribosomal and transfer RNA subunits are well known in bacteria, other gene duplication–amplification events are only now gaining attention and have probably been underreported in the literature (Andersson & Hughes, 2009; Elliott *et al.*, 2013). CNV in *msa* has been documented previously (Rhodes *et al.*, 2004), and the current study extends CNV to *p12* and *p22*.

No isolates were found to have CNV in one gene but not the others, suggesting that these genes have a functional interaction relationship in which increased copies of either are not valuable without concomitant copy number increases of the other, or that the genes are somehow duplicated together due to linkage. The latter possibility is perhaps somewhat marginalized by the known genomic distance between *msa* and *p22*, which in ATCC 33209 is around 300 000 bp between *msa1* and *p22*, going through the origin. However, it is possible that these genes are more closely located in strains other than ATCC 33209.

Although we have detected several large gene duplications, we have not been able to predict their relative orientation and distance to each other or to the rest of the chromosome. Wiens and Dale suggest a plasmid context of *msa3*, based on variable hybridization intensity in Southern blots, and another possible scenario could be the association of *msa3* with a phage, as an unconfirmed observation of an *R. salmoninarum* phage was previously reported (Fryer & Lannan, 1993). Both *msa1* and *msa2* are flanked by inverted IS sequences, notably IS*994*, and IS*3*-like insertion sequences as well as other ORFs with high homology to transposable elements and transposases, suggesting that they could be transferred and integrated through recombination or transposition mechanisms, although duplications have only been documented in *msa1* (Rhodes *et al*., 2004)

Ten of the 68 isolates screened in this study (∼15%) displayed an increased copy number *msa, p12* and *p22* genotype, in stark contrast to the 19 of 26 isolates (∼73%) that Rhodes *et al. *(2004) found to be *msa3*-positive. In their paper, every isolate except two (MT239 and GL64, which are *msa3*-negative) were from the Pacific Northwest region of the USA, suggesting that the strains circulating in that particular region have a higher frequency of multiple-copy *msa* genotypes. A predominantly North American CNV distribution is also consistent with the findings of Wiens & Dale (2008), who observed the *msa3* gene in North American but not in European isolates. In this study, the geographical origin was a much stronger predictor of CNV status than the inferred patristic distance from the phylogenetic tree. Isolates 05372K and Cow-Chs-94 for example are of lineage 1B origin, and thus thought to have diverged from lineage 1A isolates such as Carson5b between 100 and 700 years ago (Brynildsrud *et al.*, 2014). In spite of this these isolates all have duplications of the *msa, p12* and *p22* genes, a trait not shared by phylogenetic neighbours of these isolates. Note, however, that they are all sampled from fish originating from the Columbia River main basin, where multiple fish stocks co-occur. The fact that we observe this pattern of low intra-cluster but high inter-cluster patristic distances and that isolates originate from multiple geographical locations across North America (and, in a single case, Norway), sampled over a 19 year period from five different species of salmon from both freshwater and saltwater habitats, strongly suggests multiple independent introductions of the trait rather than simple inheritance.

Importantly, gene copy numbers varied widely across isolates displaying CNV. This has a number of apparent implications. Firstly, it seems that two is the basic copy number of the *msa* gene, as this genotype was by far the most common across lineages and ecosystems, was the genotype of the oldest isolates, and no isolates contained fewer than two *msa* copies. The diverse duplication pattern thus points to a base number of two *msa* genes with subsequent copy number expansions as a more parsimonious explanation than higher-value *msa* copy number and subsequent gene loss. Secondly, this heterogeneous duplication pattern indicates locally restricted gene duplication–amplification events rather than prevailing ecotypes as an explanation for the geographical clustering of CNV.

It is not clear to what extent the duplications we have found in the present work impact overall pathogen fitness. One possibility is that the observed duplications in fact represent selfish mobile genetic elements. However, this possibility contradicts the current understanding of the *msa* gene, as two copies have been proposed to confer selective advantage (O’Farrel & Strom, 1999). The immediate benefit of duplications could be through modulation of protein dosage under variable environmental conditions, while the long-term advantage is that the extra copies can, over time, accumulate mutations and evolve new functions (Conant & Wolfe, 2008; Kondrashov, 2012; Kondrashov *et al.*, 2002). In favour of a selectionist explanation is the observation that these duplications are seemingly not immediately removed from the population, but rather shared by related isolates and thus perhaps maintained in local populations. (Isolates 05372K and Cow-chs-94, for example, are closely related despite being from separate river systems and isolated 11 years apart, and they both have multiple duplications of the *msa*,* p12* and *p22* genes, although the exact numbers of each gene appear to vary.)

Rhodes *et al.* (2004) found that the presence of a third *msa* copy was clearly associated with increased mortality at lower, environmentally relevant doses. It is therefore tempting to speculate that the additional copies that we have found are increasingly beneficial to the bacterium. Such duplication–amplification events of immunomodulatory genes are now thought to be common under adaptation to new, extreme and variable environments (Elliott *et al.*, 2013), and these results point to a higher extent of such selection pressures in the Pacific Northwest than elsewhere. Our findings suggest that extra *msa* copies interact with the relatively unknown *p22* protein, as the two were always duplicated together. The nature of this interaction remains unknown and more research is needed to conclusively determine the relative fitness- and virulence relationships between different duplication-value *R. salmoninarum* isolates.

## Supplementary Data

2124 Supplementary Data
