# Supplementary material for: Identifying copy number variation of the dominant virulence factors msa and p22 within genomes of the fish pathogen Renibacterium salmoninarum
Source: Microb Genom. 2016 Apr 29;2(4):e000055. doi: 10.1099/mgen.0.000055 (PMC5320689; doi:10.1099/mgen.0.000055)
Supplement: Supplementary file 1 — Supplementary Data [file mgen-02-55-s001.pdf]

Supplementary material

Corroboration of results with real-time quantitative PCR

The rationale behind the Ct detection ratio approach is as follows: When comparing strains varying in gene copy number, the ratio of the Ct-values can quantify the relative multiplicity of a CNV gene. This would however require simultaneous detection of a ubiquitous non-CNV reference gene to minimize the influence of within-strain variation. Furthermore all primers would have to be equally efficient (and target conserved gene regions) in all target strains. The method is explained in further detail at the end of this section.

The *Renibacterium salmoninarum* strains selected for assessment by this method were 5223, Carson5b, Cow-Chs-94, 05372K, BQ96-91 and ATCC 33209 (cryopreserved as 7478 at the Norwegian Veterinary Institute). They were taken from frozen stocks kept at -80 °C, plated onto SKDM2 (1) and cultivated at 15 °C for 20 days. Genomic DNA was extracted using a Gentra Puregene Yeast/Bact. Kit (Qiagen, Hilden, Germany) according to the manufacturer's instructions. Concentration and purity of the DNA extracts were analyzed using a NanoDrop ND-1000 spectrophotometer (Thermo Scientific, Waltham, MA, USA).

Primer pairs targeting three genes, representing each major duplication cluster, were designed: Both *msa* genes (the ORFs of *msa1* and *msa2* are identical), the RSal33209_3334 gene which encodes the p22 protein, and the *lepA* gene, a ubiquitous and highly conserved housekeeping gene whose copy number is not thought to vary (2). The primers (Supplementary Table ST1) were designed in Geneious v7.1 (Biomatters,

Auckland, New Zealand) using sequences from strain ATCC 33209 as templates. A chief priority in the primer design was similar melting temperatures of all pairs.

Each qPCR reaction volume consisted of 25 µl Power SYBR Green PCR Master Mix (Applied Biosystems, Waltham, MA, USA.), 0.3 µM of both forward- and reverse primers (Invitrogen), 10 µl DNA template and a final addition of *Milli-Q* water to reach a total reaction volume of 50 µl. Subsequent qPCR was conducted on a Stratagene Mx3005P thermal cycler (Stratagene, La Jolla, CA, USA.), with thermal cycles involving i) 95°C for 10 min, ii) 45 cycles of 95°C for 15 sec; 60°C for 20 sec; 72°C for 15 sec, and iii) 95°C for 30 sec; 55°C for 30 sec; 95°C (gradual heating) for 30 sec. Fluorescence data was collected through the SYBR channel towards the end of the annealing steps (for amplification plots) and successively through the last, gradual heating step (for dissociation curves). Data analysis was performed using MxPro v4.10 software (Stratagene, La Jolla, CA, USA.).

When comparing Ct-values between strains and primer pairs, it is essential that the primer efficiencies are relatively similar both across genes and strains. This was verified by creating standard curves for each primer-and-strain pair, using four DNA-concentrations (10x dilutions). In order to minimize the influence of factors unrelated to primers and/or templates, only Ct-values from the same qPCR-setup/-run were compared in this way. We visually inspected the degree of parallelism between the standard curves, and additionally verified that the sample coefficient of variation was low (<5 %) (Fig. SF1). Under an equal efficiencies assumption, it is possible to compare Ct-values of the constant term of the standard curve directly. We enforced parallelism of the standard curves by first finding the regression line that minimized sum-of-squared

residuals for the pool of all concentration-Ct-value data points, and then proceeding with linear regression for individual isolate-gene-pairs while constraining the slope term to be equal to that found for the pooled data. This procedure finds the best-fitting parallel lines and allows direct comparison of Ct-values. Utilizing a reference gene *ref* whose copy number is (nearly) always one (such as *lepA*) to normalize within-strain variation, the copy number ratio of a gene of interest *X* between a *test* strain and a *control* strain (here; ATCC 33209), wherein the copy numbers are known *a priori*, can be found by using the following formula:

$$\frac{X_{test}}{X_{control}} = 2^{\Delta\Delta Ct} = 2^{\left(Ct_X - Ct_{ref}\right)_{control} - \left(Ct_X - Ct_{ref}\right)_{test}}$$

Here Ct_X and Ct_{ref} refer to the cycle threshold of the gene of interest and reference gene, and *test* and *control* refers to the strain of interest and control strain, respectively.

This qPCR 'comparative Ct-value' method will presumably be subject to random error and likely also unknown sources of bias, and, due to financial constraints, only a single assay was run. It should thus not be considered as entirely accurate with regards to detection of copy number variation, but rather used to corroborate results acquired e.g. through *CNOGpro* analysis, indicating whether a duplication is present or not. In silico results are not accurate either since the relationship between copy number and coverage may not in all cases be linear.

For example, in the 5223 isolate, our qPCR results indicated a per-*msa* signal strength of 1.82x baseline, or a total of 3.64 copies. The equivalent most parsimonious result of the

in silico analysis was 4 copies (obviously the true numbers are integers). For the *p22* gene, qPCR results indicated a copy number of 3.00 in 5223, while *in silico* results were most compatible with a copy number of 4 (Table ST 2). The accuracy could have been increased by performing multiple runs of sequencing and qPCR assays, respectively, but for the purposes of this paper we are satisfied with determining whether there is a duplication or not.

Phylogenetic inference

Raw reads were downloaded from the European Bioinformatics Institute using the accession numbers listed in Table 1 (Full paper.) Sequences were screened with FASTQC 0.11.3 (Andrews, 2010), quality filtered and *de novo* assembled with SPAdes 3.5.0, employing post-assembly mismatch correction (Bankevich et al., 2012; Nikolenko, Korobeynikov, & Alekseyev, 2013). Significant recombination was ruled out with SplitsTree4 (Huson, 1998). SNPs were called from the BayesHammer-corrected reads using Snippy 2.6 (Garrison & Marth, 2012; Seemann, 2014) with *R. salmoninarum* ATCC 33209 (Accession number: NC010168) as reference. Trees were built from SNP alignments using MrBayes 3.2 (Ronquist & Huelsenbeck, 2003). A Generalized Time-Reversible substitution model with uniform site distribution was used. The Markov Chain Monte Carlo settings were set to include 10,000,000 generations with subsampling every 2000th step. Burn-in was set to 1,000,000 generations. Phylogenetic trees were annotated further in FigTree 1.4.1 (http://tree.bio.ed.ac.uk/software/figtree/).

References

1. Evelyn, T., Prosperi-Porta, L., & Ketcheson J.E. (1990). Two new techniques for obtaining consistent results when growing *Renibacterium salmoninarum* on KDM2 culture medium. Dis Aquat Organ. 9:209–212.

2. Margus, T., Remm, M., & Tenson, T. 2007. Phylogenetic distribution of translational GTPases in bacteria. BMC Genomics 8:15.

3. Andrews, S. (2010). FastQC: A quality control tool for high throughput sequence data.

4. Bankevich, A., Nurk, S., Antipov, D., Gurevich, A. A., Dvorkin, M., Kulikov, A. S., Lesin, V.M., Nikolenko, S. I., Pham, S., & Prjibelski, A. D. (2012). SPAdes: a new genome assembly algorithm and its applications to single-cell sequencing. *Journal of Computational Biology, 19*(5), 455-477.

5. Garrison, E., & Marth, G. (2012). Haplotype-based variant detection from short-read sequencing. *arXiv preprint arXiv:1207.3907*.

6. Huson, D. H. (1998). SplitsTree: analyzing and visualizing evolutionary data. *Bioinformatics, 14*(1), 68-73.

7. Nikolenko, S. I., Korobeynikov, A. I., & Alekseyev, M. A. (2013). BayesHammer: Bayesian clustering for error correction in single-cell sequencing. *BMC genomics, 14*(1), 1.

8. Ronquist, F., & Huelsenbeck, J. P. (2003). MrBayes 3: Bayesian phylogenetic inference under mixed models. *Bioinformatics, 19*(12), 1572-1574.

9. Seemann, T. (2014). Snippy. https://github.com/tseemann/snippy.

Target gene	Primer name	Sequence (5' - 3')	Length	Mol. wt.	Tm
msa	MSA1_1507F	GATGCCCAGACTGTTGCCT	19	5780.8	70
	MSA1_1611R	CTCAAAAACACCGAAACTCGTCTTA	25	7564.0	71
p22	p22_140F	AGAACACTTCTGACTTTGTGGTAGATA	27	8314.4	72
	p22_234R	GCTTGCTTGGTTGAGCGTAAA	21	6493.2	69
lepA	lepA_1490F	CGGATCTGGTCAAGGTCGATATT	23	7095.6	72
	lepA_1604R	CGCAATTTCCCCGTCATCATC	21	6278.2	71

Table ST 1 Primers used for qPCR corroboration

Isolate	Accession	# *msa*		# *p22*	
		Cov.	qPCR	Cov.	qPCR
05372K	ERR327906	4.87	4.10	4.12	3.03
5223	ERR327964	4.43	3.64	3.58	3.00
BQS96_91-1	ERR327963	3.53	2.20	2.15	1.44
Carson5b	ERR327905	5.28	5.00	4.32	4.50
Cow_Chs_94	ERR327915	3.29	3.50	2.22	2.80
7478 / ATCC 33209	NC_010168.1	2.24	2	1.20	1

Table ST 2 Correspondence between copy numbers as inferred through read coverage and qPCR.

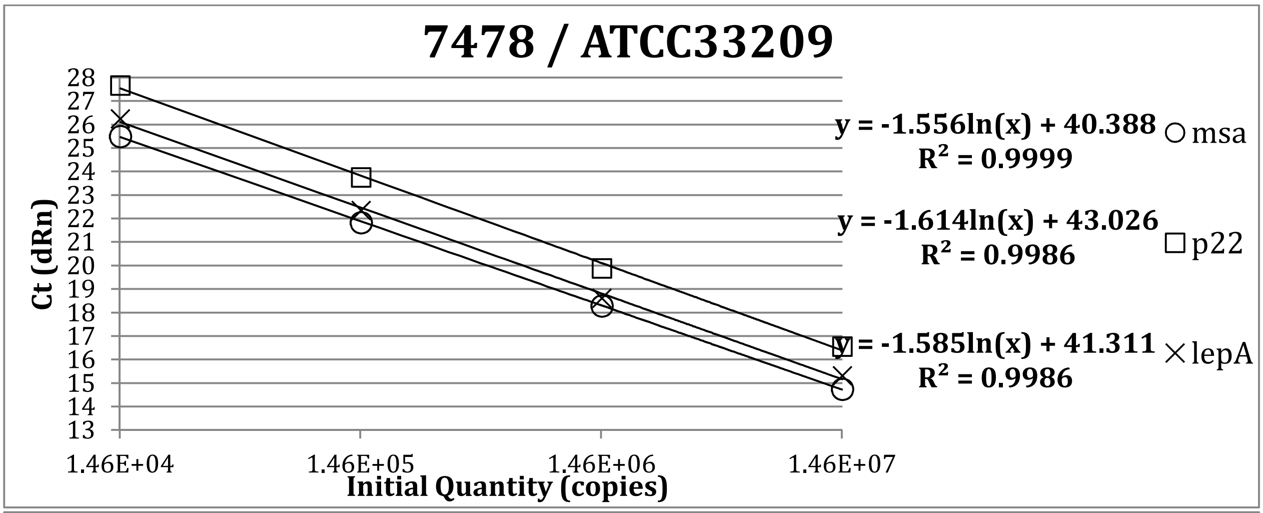
7478 / ATCC33209
Ct (dRn)
Initial Quantity (copies)
1.46E+04
1.46E+05
1.46E+06
1.46E+07
y = -1.556ln(x) + 40.388
R² = 0.9999
y = -1.614ln(x) + 43.026
R² = 0.9986
y = -1.585ln(x) + 41.311
R² = 0.9986
msa
p22
lepA

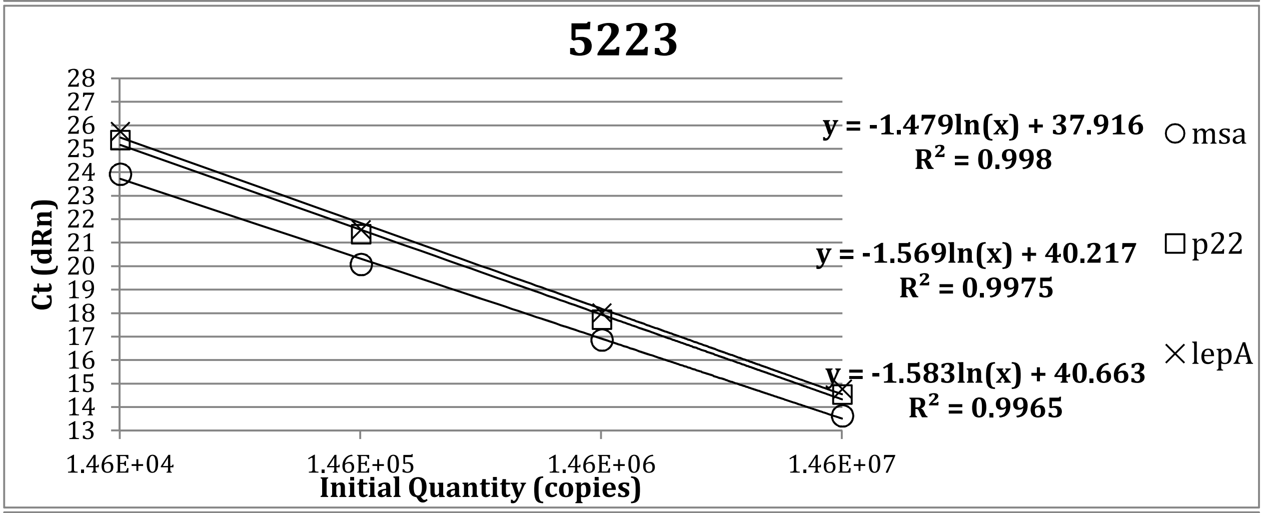
5223
Ct (dRn)
Initial Quantity (copies)
1.46E+04
1.46E+05
1.46E+06
1.46E+07
y = -1.479ln(x) + 37.916
R² = 0.998
y = -1.569ln(x) + 40.217
R² = 0.9975
y = -1.583ln(x) + 40.663
R² = 0.9965
msa
p22
lepA

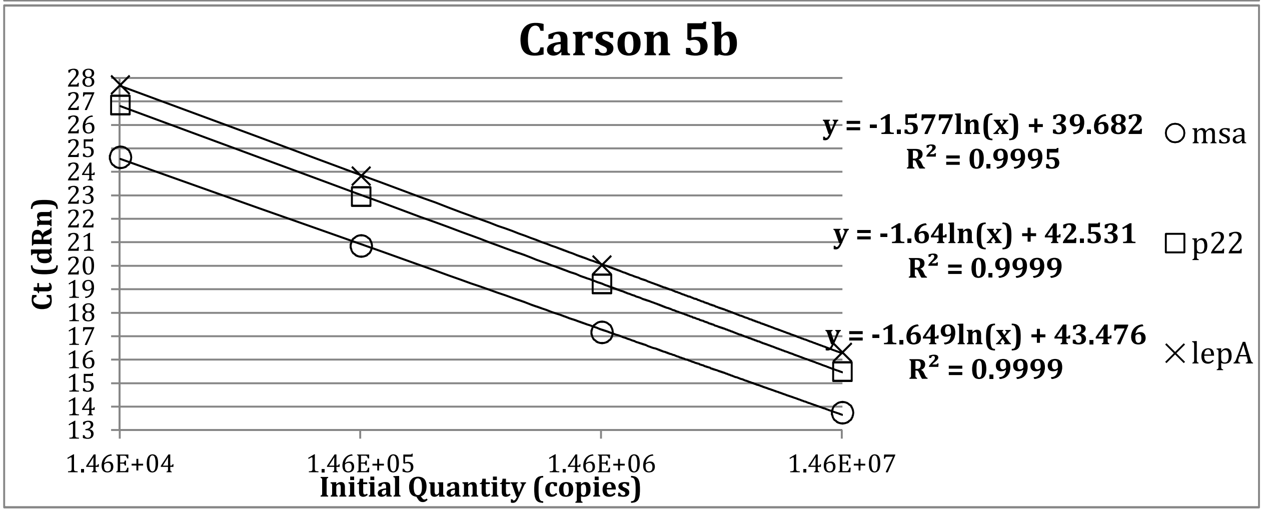
Carson 5b
Ct (dRn)
Initial Quantity (copies)
1.46E+04
1.46E+05
1.46E+06
1.46E+07
y = -1.577ln(x) + 39.682
R² = 0.9995
y = -1.64ln(x) + 42.531
R² = 0.9999
y = -1.649ln(x) + 43.476
R² = 0.9999
msa
p22
lepA

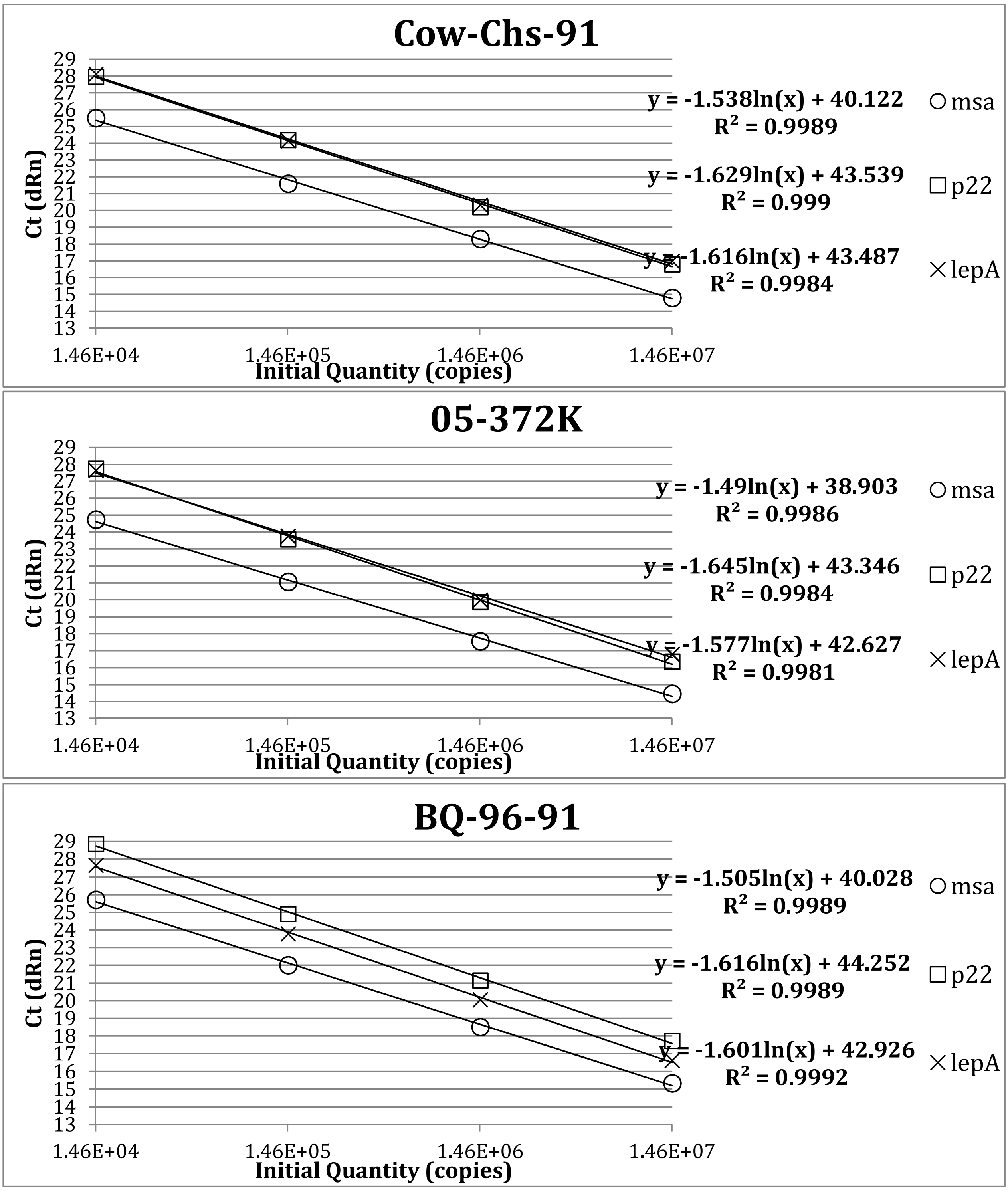

Fig. SF 1 Standard curves for the reference strain (7478 / ATCC 33209) and selected CNV-positive strains.
